# Quinoxaline protects zebrafish lateral line hair cells from cisplatin and aminoglycosides damage

**DOI:** 10.1038/s41598-018-33520-w

**Published:** 2018-10-11

**Authors:** Sonia M. Rocha-Sanchez, Olivia Fuson, Shikha Tarang, Linda Goodman, Umesh Pyakurel, Huizhan Liu, David Z. He, Marisa Zallocchi

**Affiliations:** 1 School of Dentistry, Creighton University, Omaha, NE 68178 USA; 2 Boys Town National Research Hospital, Omaha, NE 68131 USA; 3 Department of Biomedical Sciences, Creighton University School of Medicine, Omaha, NE 68178 USA

**Keywords:** Hair cell, Inner ear

## Abstract

Hair cell (HC) death is the leading cause of hearing and balance disorders in humans. It can be triggered by multiple insults, including noise, aging, and treatment with certain therapeutic drugs. As society becomes more technologically advanced, the source of noise pollution and the use of drugs with ototoxic side effects are rapidly increasing, posing a threat to our hearing health. Although the underlying mechanism by which ototoxins affect auditory function varies, they share common intracellular byproducts, particularly generation of reactive oxygen species. Here, we described the therapeutic effect of the heterocyclic compound quinoxaline (Qx) against ototoxic insults in zebrafish HCs. Animals incubated with Qx were protected against the deleterious effects of cisplatin and gentamicin, and partially against neomycin. In the presence of Qx, there was a reduction in the number of TUNEL-positive HCs. Since Qx did not block the mechanotransduction channels, based on FM1-43 uptake and microphonic potentials, this implies that Qx’s otoprotective effect is at the intracellular level. Together, these results unravel a novel therapeutic role for Qx as an otoprotective drug against the deleterious side effects of cisplatin and aminoglycosides, offering an alternative option for patients treated with these compounds.

## Introduction

Unlike other sensory receptors, normally present in millions, the number of mammalian auditory hair cells (HCs) is significantly smaller^1,2^. Their limited number and extreme vulnerability to environmental and genetic insults, as well as their inability to spontaneously regenerate after damage dictates that loss of only a fraction of HCs is sufficient to cause a significant and irreversible hearing deficit^3,4^. In fact, hearing loss is one of the most common chronic conditions affecting individuals of all ages, ethnicities, and genders. Currently, 15% of US adults ages 18–64 and 28% of people over 65 have some level of hearing impairment, varying from moderate to complete deafness in one or both ears^5–8^.

Ototoxic drugs such as heavy metals, alkaloids, aminoglycoside antibiotics and platinum-based drugs are among the external insults that can lead to the loss of sensory HCs^4,9–20^. Gentamicin and neomycin, two commonly use aminoglycosides against Gram-negative bacterial infections^9–11^, can cause HC death via activation of programmed cell death pathways^13–16^. However, the ototoxic damage mediated by these two drugs follows different time courses, suggesting a differential regulation of HC death mechanisms^21,22^. On the other hand, cisplatin is a highly ototoxic chemotherapeutic platinum-derivative drug that can damage and kill HCs^23–28^. Both, aminoglycosides’ and cisplatin’s ototoxic effects are associated with reactive oxygen species (ROS) accumulation^4,21,23,29–31^.

Since the discovery of the toxic side effects of cisplatin and aminoglycosides to the inner ear, several studies have been directed towards the understanding of drug-mediated HC death. Most importantly, these studies have highlighted the need for pharmacological interventions to protect hearing, particularly, the search for compounds that will result in inhibition of HC apoptosis^32^. While several clinical trials are underway, experience tells us that not all patients will respond the same way to even the most successful agent, which provides clues to the need for different alternative approaches to address this problem. Indeed, any effective otoprotective strategy will require intervention at several levels along the pathways regulating cell death and survival^33^.

Zebrafish HCs from the lateral line are structurally, functionally and molecularly similar to mammalian inner ear HCs^16,29,34,35^. Among many common features, mammalian and zebrafish HCs display a polarized apical hair bundle with mechanotransduction capabilities, extracellular tip links involved in the gating of the mechanotransduction channels and ribbon synapses^16,29,34^. Similarly to mammalian HCs, zebrafish HCs die in response to ototoxic drugs including cisplatin and aminoglycosides, making them an excellent model for investigating potentially otoprotective agents^20–23,35^. One key difference is that contrary to mammalian HCs, zebrafish HCs can regenerate after trauma through the proliferation of their associated supporting cells^22,36–38^.

Several studies have shown the potential otoprotective effect of quinoline ring derivatives against aminoglycosides^22,39^. In this report, we describe the therapeutic effect of the heterocyclic compound quinoxaline (Qx), a small quinoline ring derivative, against cisplatin and aminoglycosides in zebrafish HCs. Qx has manifold properties, including neuroprotective^40^, antimicrobial^41^, antitumoral^42,43^, antitrypanosomal^44^, anti-inflammatory/antioxidant activities^45^, and immunoregulation^46,47^. Qx’s diversity of activities seems to be dependent on its metabolization, dosage, and length of treatment^48,49^. In HCs from the zebrafish lateral line, Qx confers protection against the harmful effect of cisplatin and gentamicin and partially against neomycin. HC mechanotransduction activity was normal in Qx treated animals, suggesting that Qx is not blocking ototoxin uptake by the HCs but its harmful intracellular effects. Qx did not seem to exert any direct effect on HC proliferation. However, we observed an increase in the number of BrdU-positive supporting cells, suggesting that, over time, Qx might be stimulating the generation of new HCs through supporting cell proliferation and differentiation. Overall, the present study supports a novel role for Qx in HC protection in the zebrafish lateral line and underscore its potential use in the treatment of drug-induced hearing loss.

## Results

### Quinoxaline protects HCs against cisplatin

**Figure 1:**
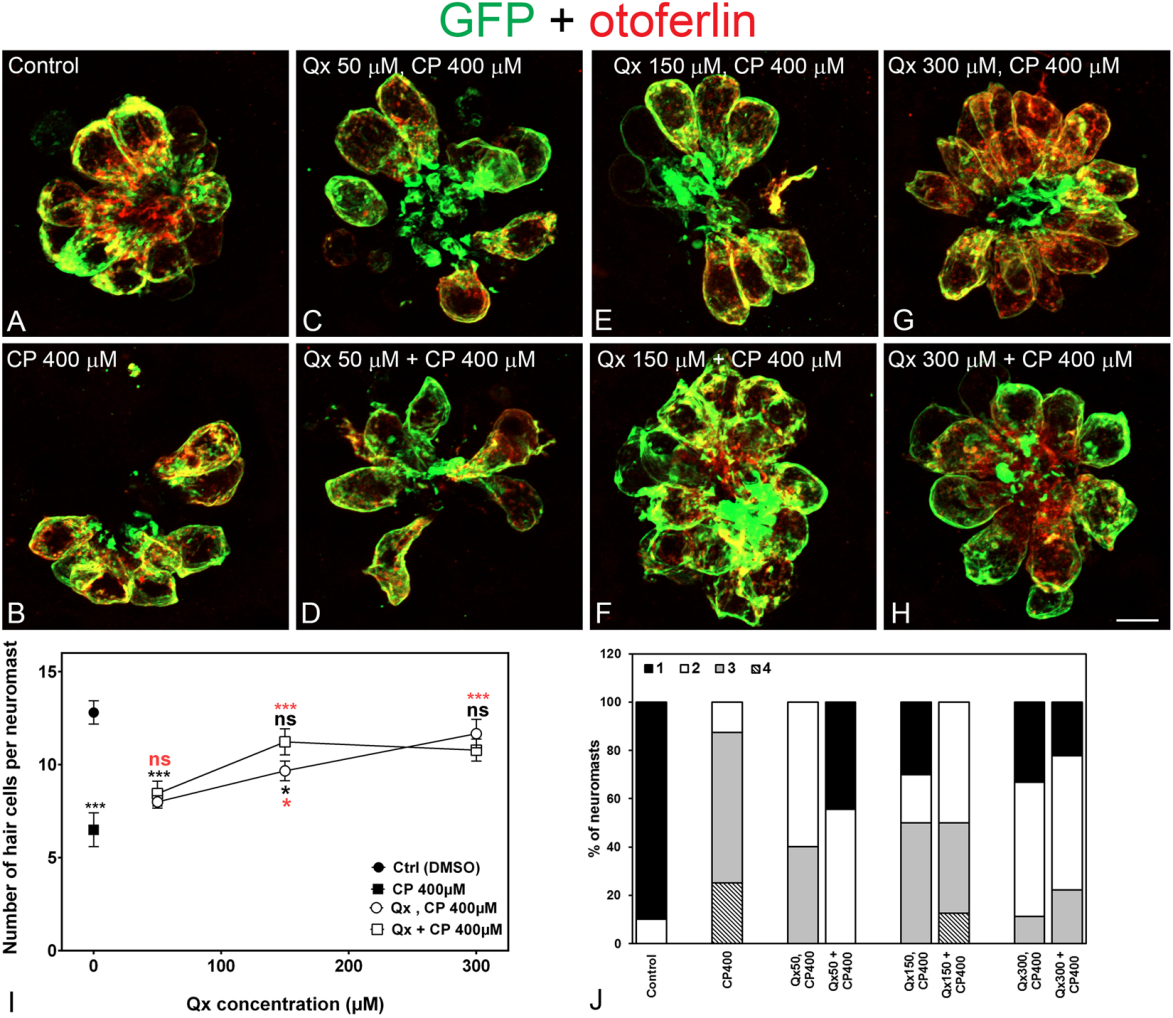
Qx protects against cisplatin ototoxicity. 5dpf Tg(brn3c:GFP) larvae were incubated with vehicle alone (DMSO, **A**), 400 μM of cisplatin (CP, **B**), pre-treated with Qx for 2 hours and then incubated with CP for 6 hours (Qx, CP, **C**,**E**,**G**) or pre-treated with Qx for 2 hours and then co-treated with Qx and CP for 6 more hours (Qx + CP, **D**,**F**,**H**). Animals were fixed and immunostained for GFP (green) and otoferlin (red). (**I**) Quantification of the number of hair cells per neuromast after the different treatments represented as mean +/− SEM. One-way ANOVA, Dunnett post test. *p < 0.05, ***p < 0.001. Black asterisks compared *versus* control. Red asterisks compared *versus* CP 400 µM. (**J**) Scores for neuromast morphology (see Materials and Methods). Scale bar: 6 μm. Data were taken from at least 20 animals and 3 experiments runs.

We first tested whether Qx protects zebrafish HCs of the lateral line from cisplatin (CP) toxicity and if this protection was dose-dependent (Fig. 1). Two incubation protocols were employed. Protocol 1: 5dpf (days post-fertilization) zebrafish larvae were pre-incubated with Qx (50–300 μM) for 2 hours, followed by 6 hours incubation with 400 μM of CP (Qx, CP). Protocol 2: 5dpf larvae were pretreated with Qx for 2 hours, followed by a 6 hours co-incubation with Qx and CP (Qx + CP). Larvae treated with CP alone showed a significant HCs loss (~50%, Fig. 1B,I) compared to DMSO (control) treated animals (Fig. 1A,I). No differences in HC numbers were observed between CP alone, and the lowest dose of Qx (50 μM) employed in our studies for any of the incubation protocols (Fig. 1C,D,I). However, when fish were exposed to 300 μM of Qx, we observed full protection against CP with both incubation protocols, suggesting this is the optimal protective dose against cisplatin-induced hair cell death (Fig. 1G–I). At the intermediate Qx concentration (150 μM) we observed variable results depending on the incubation protocol employed (Fig. 1E,F,I). Scores for neuromast morphology (see Material and Methods for the explanation of score assignments) showed an improvement in neuromast gross morphology when cisplatin-exposed larvae were treated with the different Qx concentrations (Fig. 1J).

**Figure 2:**
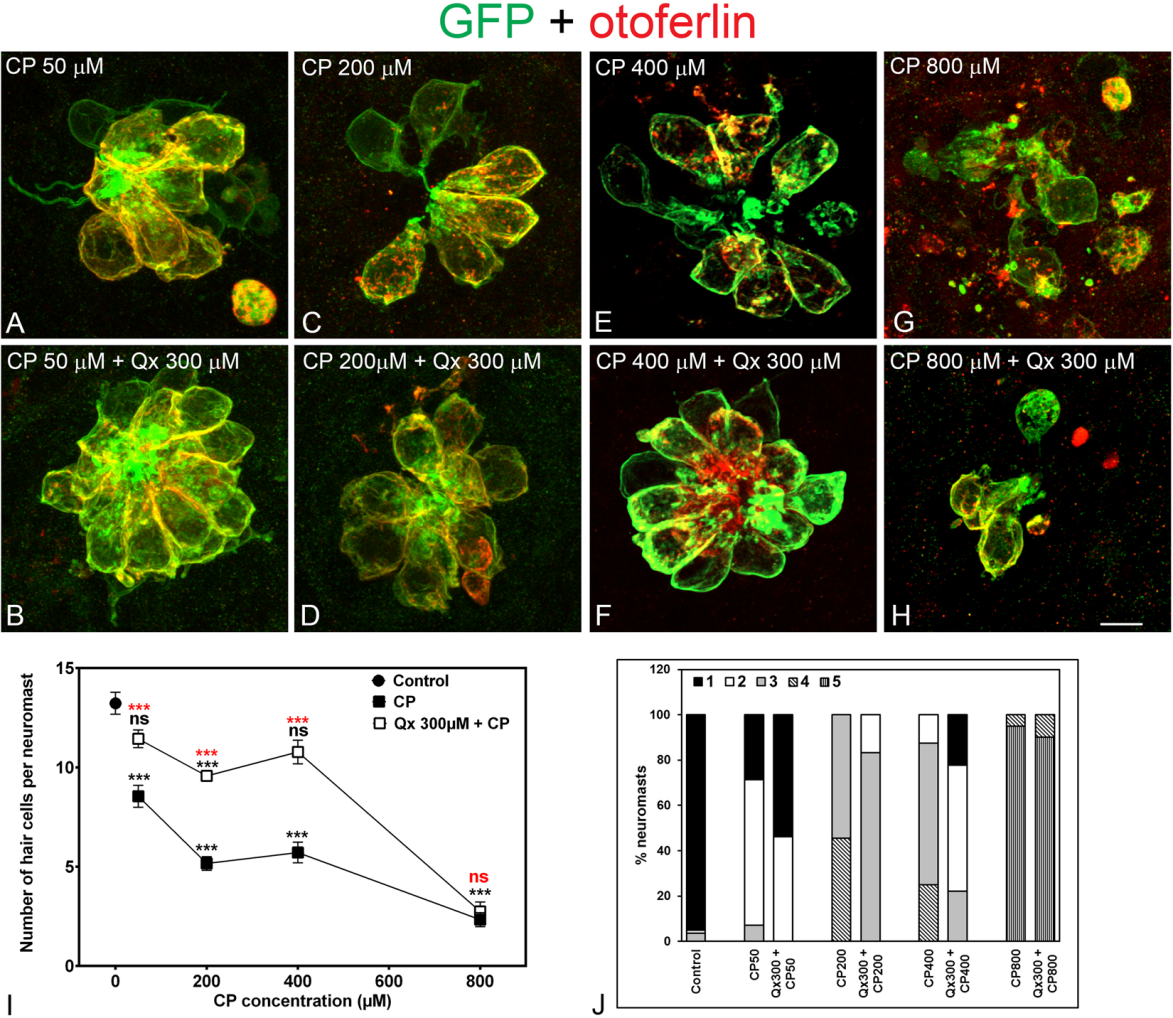
Dose protection curve against CP. 5dpf Tg(brn3c:GFP) larvae were incubated with 50 µM to 800 µM of CP (**A**,**C**,**E**,**G**) for 6 hours or pre-treated with 300 µM of Qx for 2 hours and then co-treated with Qx and CP (50µM-800µM) for 6 hours (**B**,**D**,**F**,**H**). Animals were fixed and immunostained for GFP (green) and otoferlin (red). Control animals were exposed to vehicle alone (DMSO). (**I**) Quantification of the number of hair cells per neuromast after the different treatments represented as mean +/− SEM. One-way ANOVA, Dunnett post test. ***p < 0.001. Black asterisks compared *versus* DMSO-treated animals. Red asterisks compared *versus* the corresponding CP concentration. (**J**) Scores for neuromast morphology (see Materials and Methods). Scale bar: 6 μm. Data were taken from at least 20 animals and 3 experiments runs.

Having established the Qx protective dose against CP, we determined the dose-response relationship by analyzing the number of HCs after exposure to different concentrations of CP (50–800 µM) in the presence or absence of Qx 300 µM (Fig. 2). Incubation conditions were as indicated for Protocol 2. We observed significant Qx’s protection against CP concentrations ranging from 50 µM to 400 µM (Fig. 2A–F,I). When animals were incubated with Qx and 50 µM or 400 µM of CP the number of HCs per neuromast reached the control values. QX + CP 200 µM treatment also showed HC protection, but it never reached the control values. Animals exposed to higher CP concentrations (800 µM) did not show any protection in the presence of Qx (Fig. 2G–I). Neuromast gross morphology was improved in the presence of Qx (Fig. 2J) for the CP doses between 50µM-400µM, suggesting that Qx not only protects HC from CP but also preserves neuromast gross morphology.

Overall these results suggest Qx protects hair cells from the toxic side effect of cisplatin.

### Quinoxaline’s effect on aminoglycoside-mediated ototoxicity

**Figure 3:**
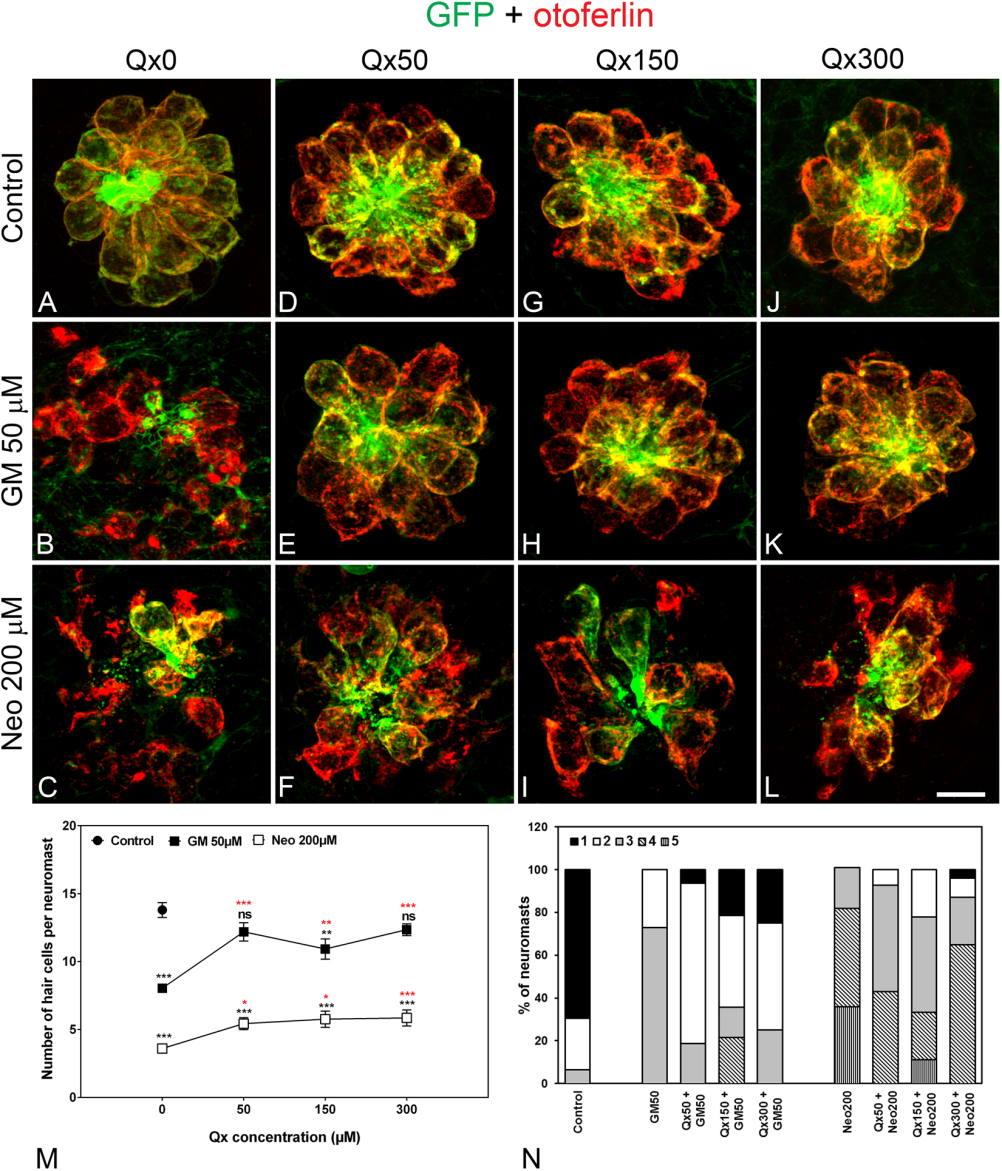
Dose protection curve against aminoglycosides. 5dpf larvae were incubated with vehicle (E3, **A**) or with 50 μM (**D**–**F**), 150 μM (**G**–**I**) or 300 μM (**J**–**L**) of Qx for a total of 8 hours. Gentamicin (GM, 50 μM, **B**,**E**,**H**,**K**) or neomycin (Neo, 200 µM,**C**,**F**,**I**,**L**) were added during the last 60 min or 30 min of incubation, respectively. Animals were fixed and stained for otoferlin (red) and GFP (green). (**M**) Quantification of the number of hair cells per neuromast after the different treatments represented as mean +/− SEM. Note that since no significant differences were found in the number of hair cells per neuromast when animals were incubated with the different Qx concentrations (0–300 µM, **A**,**D**,**G**,**J**), the control value represents the average of all these treatments. One-way ANOVA, Dunnett post test. *p < 0.05, **p < 0.01, ***p < 0.001. Black asterisks compared *versus* control. Red asterisks compared *versus* the corresponding aminoglycoside-only treatment. (**N**) Scores for neuromast morphology (see Materials and Methods). Scale bar: 7 μm. Data were taken from at least 20 animals and 3 experiments runs.

We then examined Qx’s potential otoprotective effect against gentamicin (GM) and neomycin (Neo), two antibiotics clinically used against a broad spectrum of bacterial infections^9^. Dose-response curves for Qx were performed in the presence of 50 μM of GM or 200 μM of Neo (Fig. 3). Pre-treatment of zebrafish larvae with Qx (50–300 μM), followed by co-treatment with Qx + GM (Fig. 3B,E,H,K,M) showed protection of neuromast HCs for all the Qx concentrations tested. No significant differences in the number of HCs were observed between animals co-treated with GM and Qx 50 μM or 300 μM (Fig. 3E,K,M) and the corresponding controls (Fig. 3D,J,M), demonstrating complete protection from GM-induced HC death. In the case of Qx’s intermediate dose (150 μM) (Fig. 3H,M), we observed some variability with only 80% of HC protection compared to Qx-only treated animals (Fig. 3G,M).

Next, we studied Qx’s potential otoprotective effect against Neo. Pre-treatment of zebrafish larvae with 50 μM to 300 μM Qx, followed by co-treatment with 200 μM of Neo and Qx, partially protected HCs from ototoxic damage (Fig. 3F,I,L,M). Regardless of the Qx’s concentration tested, we always observed approximately 45% of HC loss compared to control animals (Fig. 3A,D,G,J,M). Nevertheless, the number of HCs retained in Qx-treated larvae was significantly higher than that of Neo alone treated animals (Fig. 3C,M).

Neuromast scores (Fig. 3N) reflected some improvement in neuromast gross morphology when animals were co-treated with the corresponding aminoglycoside and Qx, with the highest Qx concentration (300 μM) showing better scores. These final results suggest that Qx not only protects HCs from GM and Neo but also that Qx helps to preserve neuromast morphology.

No differences were observed in HC numbers or neuromast scores between vehicle-treated animals and animals exposed to any of the Qx concentrations (Fig. 3A,D,G,J).

**Figure 4:**
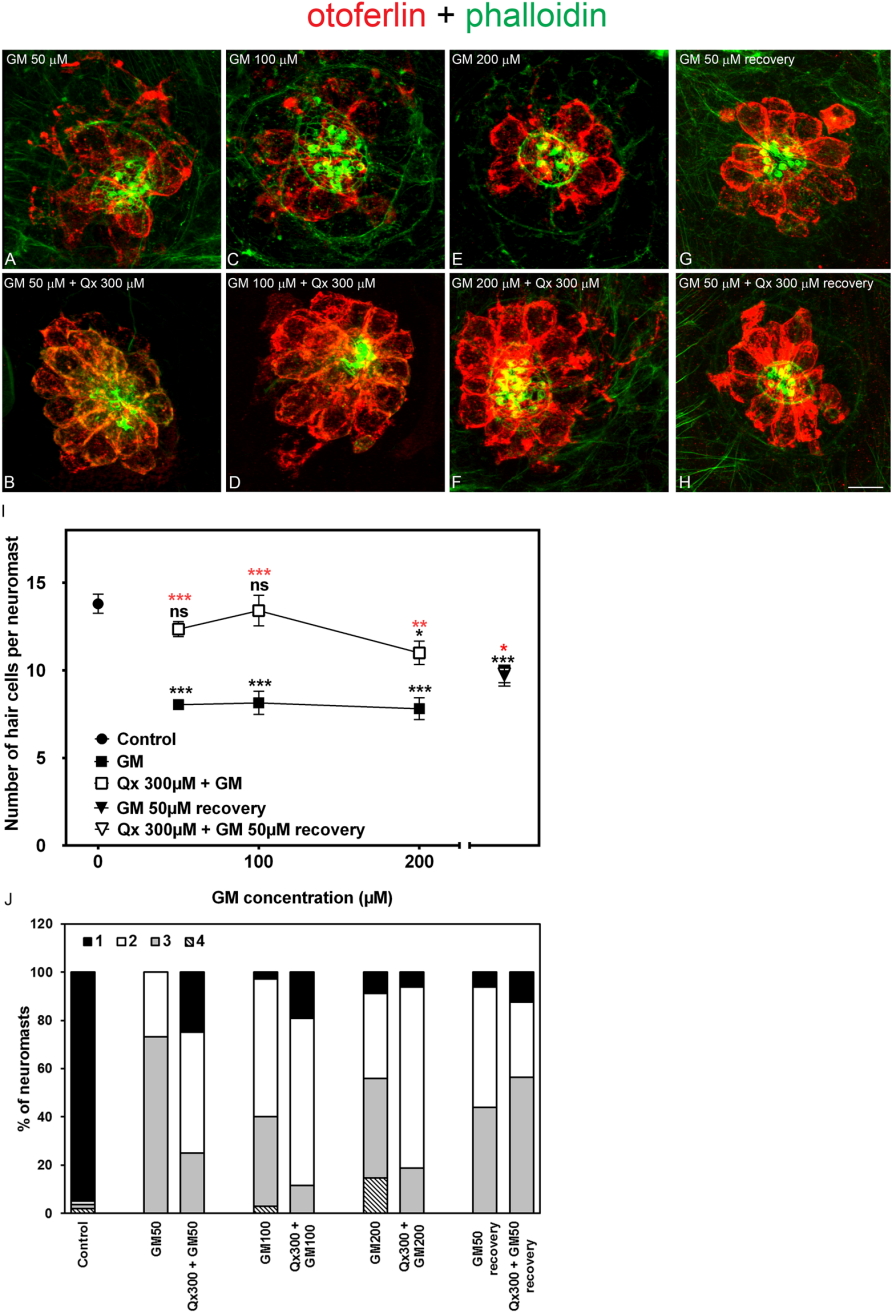
Qx protects against the acute phase of gentamicin ototoxicity. 5dpf zebrafish were treated for 60 min with gentamicin (GM) 50 μM-200 μM (**A**,**C**,**E**,**G**) or pre-treated for 7 hours with Qx 300 μM followed by co-treatment with Qx and gentamicin for 1 more hour (**B**,**D**,**F**,**H**). Untreated or Qx-treated animals were used as controls. (**G**,**H**) Animals treated with GM 50 µM, with or without 300 µM of Qx were transferred for 5 hours to fresh E3 media before fixation. Fixed animals were immunostained for otoferlin (red), counterstained with phalloidin (green) and analyzed by confocal microscopy. (**I**) Quantification of the number of hair cells per neuromast after the different treatments represented as mean +/− SEM. One-way ANOVA, Dunnett post test. *p < 0.05, **p < 0.01, ***p < 0.001. Black asterisks compared *versus* control. Red asterisk compared *versus* the corresponding GM-only treatment. For recovery experiments: Black asterisk compared *versus* control animals, red asterisk compared *versus* GM-only treatment without recovery. (**J**) Scores for neuromast morphology. Scale bar: 7 μm. Data were taken from at least 20 animals and 3 experiments runs.

**Figure 5:**
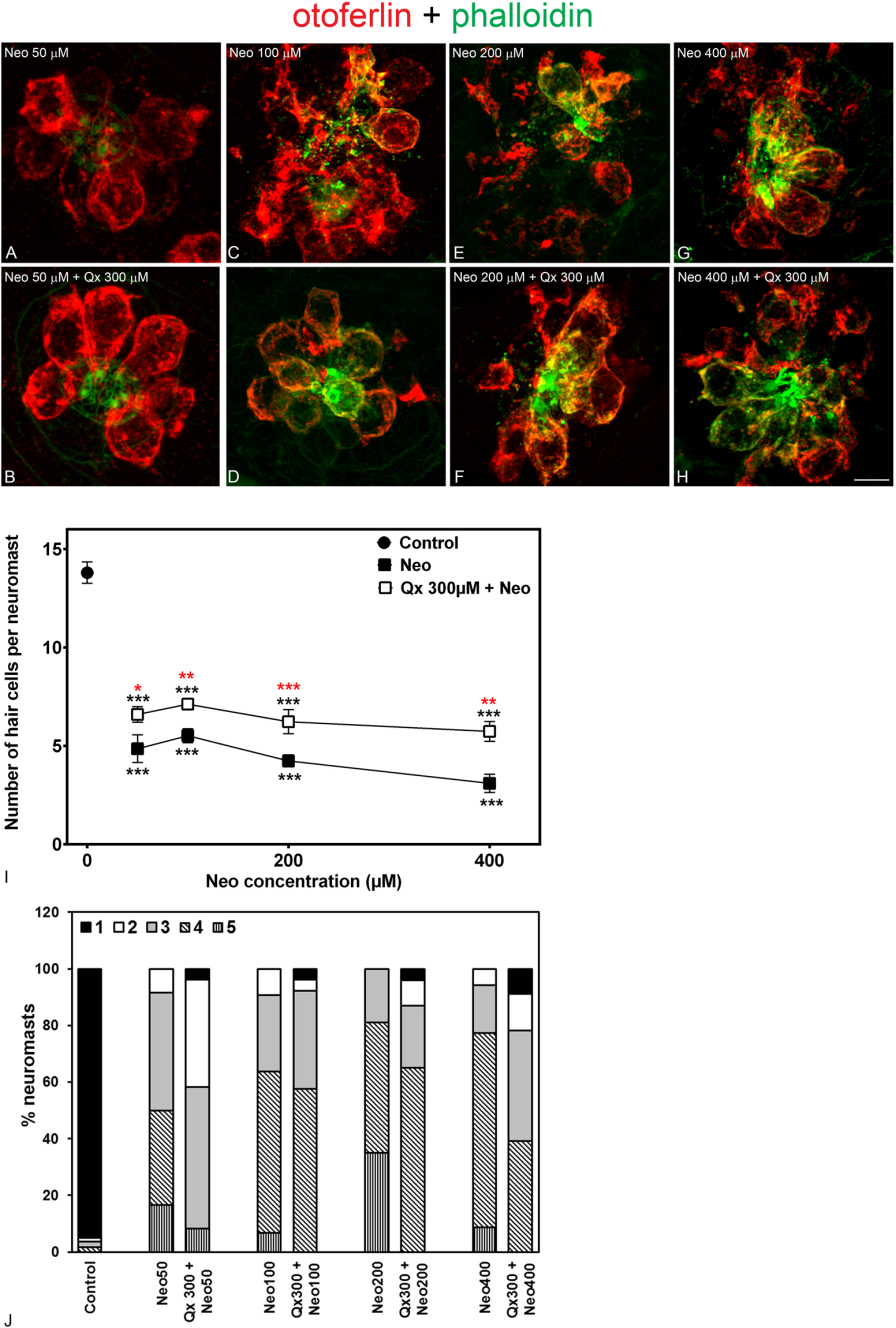
Qx partially protects against acute exposure to neomycin. 5dpf larvae were treated for 30 min with neomycin (Neo) 50 μM-400 μM (**A**,**C**,**E**,**G**) or for 8 hours with Qx 300 μM and Neo was added 30 min before the end of the incubation time (**B**,**D**,**F**,**H**). Untreated or Qx-treated animals were used as controls. Animals were fixed, stained for otoferlin (red) and phalloidin (green) and analyzed by confocal microscopy. (**I**) Quantification of the number of hair cells per neuromast after the different treatments represented as mean +/− SEM. One-way ANOVA, Dunnett post test. *p < 0.05, **p < 0.01, ***p < 0.001. Black asterisks compared *versus* control. Red asterisk compared *versus* the corresponding Neo-only treatment. (**J**) Scores for neuromast morphology. Scale bar: 6 μm. Data were taken from at least 20 animals and 3 experiments runs.

Based on the previous results (HC protection and neuromast scores), we chose the Qx concentration of 300 μM as the optimal otoprotective dose to analyze its effect against a range of aminoglycoside concentrations (Figs 4 and 5). When zebrafish larvae were incubated with a range of GM concentrations ranging from 50 μM to 200 μM, we observed a significant increase in HC number in the presence of Qx compared to GM alone (Fig. 4A–F,I). While there was full protection for the low doses of GM (50–100 μM) with values similar to vehicle-treated animals, we only attained 80% protection when fish were incubated with the highest GM doses (200 μM). Neuromast scores showed a tendency towards normal morphology with Qx treatment, although they never reached the control values (Fig. 4J).

Previous work has shown that gentamicin’s ototoxicity can be divided into at least two phases, a rapid phase occurring over a period of 30 to 90 min and a more slowly cumulative phase that continues, even in the absent of gentamicin, for 3 to 6 hours^21^. Based on this, we decided to address whether Qx also protects from GM’s long-term ototoxic effect. Animals were exposed to GM (50 µM) alone or in the presence of Qx as described above followed by a recovery period of 5 hours in fresh E3 media (Fig. 4G–I). Animals treated with GM alone showed a modest albeit significant improvement in HC number after the 5 hours recovery, compared to GM-exposed animals with no recovery. However, HC number never reached the control values. Similar results were obtained with the animals co-treated with Qx. The fact that we obtained comparable HC protection with or without Qx suggests that Qx is preventing GM’s acute ototoxic effect but not the long-term GM’s cumulative toxicity. The overall neuromast morphology was improved after the 5 hours recovery but never reached control values (Fig. 4J).

A similar set of experiments was performed for neomycin with a concentration range between 50 μM to 400 μM (Fig. 5). These experiments showed that Qx was only able to protect between 40–50% of the neuromast HCs (Fig. 5A–I), suggesting this is the maximum protection that Qx can provide against Neo under any condition. Similar to GM-treated animals, neuromast scores never reached the controls values, although they showed a tendency towards a better morphology when Qx was present (Fig. 5J).

Collectively the data demonstrate that Qx offered protection from gentamicin- and neomycin-ototoxicity, albeit with different levels of efficacy.

### Quinoxaline prevents hair cell death

**Figure 6:**
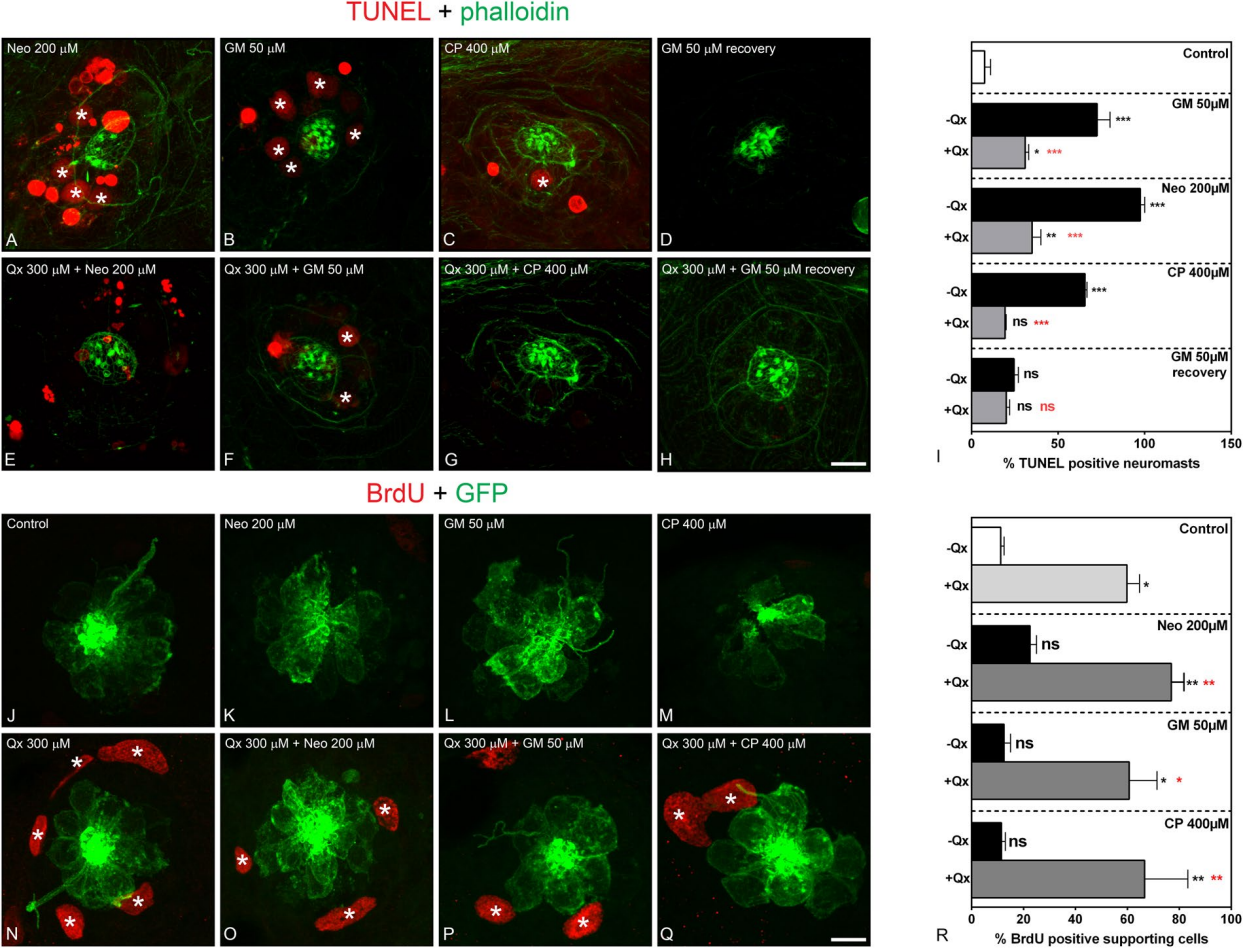
Qx protects against ototoxin-induced HC death and promotes supporting cell proliferation. (**A**–**I**) TUNEL assay (red) was performed in zebrafish incubated with vehicle or 300 µM of Qx alone (controls) or with the corresponding ototoxin with or without Qx 300 µM. Animals were counterstained with phalloidin (green). (**A**) Neomycin (Neo) 200 µM incubation for 30 min. (**B**) Gentamicin (GM) 50 µM incubation for 1 hour. (**C**) Cisplatin (CP) 400 µM incubation for 2 hours. (**D**) GM 50 µM incubation for 1 hour followed by recovery for 5 hours. (**E**) Incubation with Qx 300 µM for 8 hours and Neo 200 µM for 30 min. (**F**) Incubation with Qx 300 µM for 8 hours and GM 50 µM for 1 hour. (**G**) Qx 300 µM for 8 hours + CP 400 µM for 2 hours. (**H**) Qx 300 µM incubation for 8 hours + GM 50 µM for 1 hour followed by 5 hours recovery. Asterisks denote TUNEL-positive HCs. (**I**) The percentage of TUNEL-positive neuromasts was calculated for each treatment and represented as mean +/− SEM. (**J**–**R**) Proliferation assays were performed in 5dpf *Tg(brn3c:GFP)* in the presence/absence of Qx and the corresponding ototoxin, by the BrdU-labelling method (red). Animals were immunostained for GFP (green). (**J**) control. (**K**) Neo 200 µM 30 min. (**L**) GM 50 µM 1 hour. (**M**) CP: 400 µM 2 hours. (**N**) Qx 300 µM 8 hours. (**O**) Qx 300 µM 8 hours + Neo 200 µM 30 min. (**P**) Qx 300 µM 8 hours + GM 50 µM 1 hour. (**Q**) Qx 300 µM 8 hours + CP 400 µM 2 hours. Asterisks denote neuromast supporting cells positive for BrdU. (**R**) The percentage of BrdU-positive supporting cells per neuromast was calculated for each treatment and represented as mean +/− SEM. One-way ANOVA, *p < 0.05, **p < 0.01. Black asterisks compared *versus* corresponding control. Red asterisk compared *versus* the corresponding ototoxin-only treatment. Scale bar: (**A**–**H**) 10 µm, (**J**–**O**) 7 μm. Data were taken from at least 15 animals and 3 experiments runs.

To determine whether Qx prevents ototoxin-induced HC death or whether it promotes HC proliferation, we conducted terminal deoxynucleotidyl transferase dUTP nick-end labeling (TUNEL) and HC proliferation experiments, respectively. Animals were incubated with the ototoxin in the presence/absence of 300 μM of Qx, and the number of neuromasts showing TUNEL-positive HCs quantified and compared to vehicle- and ototoxin-only -treated animals. Similar to previous studies^30,50,51^, exposure to aminoglycosides or cisplatin alone resulted in a significant increase in the number of TUNEL-positive neuromasts (Fig. 6A–C,I). When larvae were co-treated with 300 μM of Qx, we observed a significant reduction in HC death (Fig. 6E–G,I), further supporting Qx’s protective effects against toxin-induced HC death. In the case of aminoglycoside protection, although this reduction in TUNEL-positive neuromasts was significant compared to the ototoxin alone, it never reached control values (vehicle-treated animals). When animals treated with GM, with or without Qx, were left for 5 hours in E3 media, no TUNEL-positive neuromasts were observed (Fig. 6D,H,I), suggesting that the HCs, although still in reduced number compared to controls (Fig. 4I) are in the process of recovering from the harmful ototoxin treatment. Again, this latter result supports the notion of Qx only acting during the acute GM’s toxic effect.

Unlike mammalian HCs, non-mammalian HCs can quickly regenerate after ototoxic damage^36–38^. We, therefore, determined whether HC regeneration was involved in Qx-mediated otoprotection by performing cell proliferation experiments using BrdU. Animals were treated with the different ototoxins (400 μM CP, 50 μM GM or 200 μM Neo) and BrdU in the presence/absence of Qx 300 μM, and BrdU incorporation analyzed by immunohistochemistry (Fig. 6J–R). As expected from previous work^22,37^, no BrdU-positive HCs were observed during these short incubation times, suggesting Qx does not induce HC proliferation under these conditions. However, Qx was able to stimulate the proliferation of supporting cells, determined by BrdU positive immunoreactivity (Fig. 6N–R). This last result was comparable to the one obtained by Kruger *et al*.^22^ when performing BrdU incubations over short periods of time and, in our case, reinforces the idea of Qx’s diversity of activities depending on its specific intracellular metabolization^48,49^.

Overall, the data demonstrate that Qx protects HCs from cisplatin and aminoglycosides damage by preventing HC death rather than by promoting HC proliferation.

### HC mechanotransduction activity is not impaired by quinoxaline treatment

**Figure 7:**
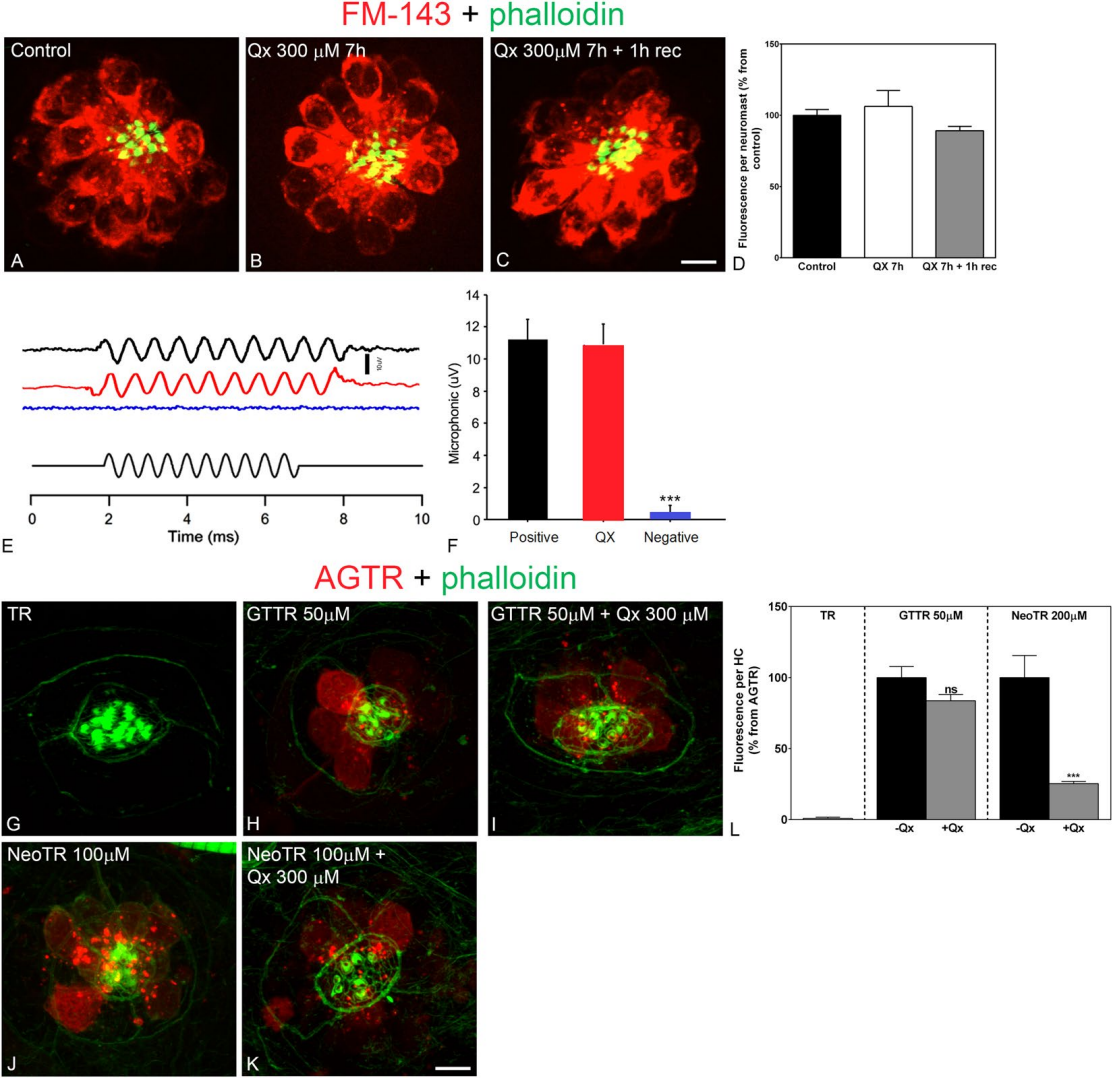
Treatment with Qx does not abolish mechanotransduction channel activity. 5dpf wild type larvae were incubated in E3 media (**A**), Qx 300 μM for 7 hours and immediately assayed for FM1-43 uptake (**B**) or Qx, 300 µM, recovered for 1 hour and then treated with the dye (**C**). Quantification of the fluorescent intensity per neuromast (**D**) was expressed as percentage from controls. No significant differences were observed between control and treated animals (unpaired Student’s t-test). (**E**,**F**) Microphonic potentials from animals treated with vehicle (black) or 300 μM of Qx (red). Pcdh15a mutants were used as a negative control (blue). Microphonic responses are represented as mean +/− SD. Only the Pcdh15a mutants showed a significant decrease in microphonic potentials compared to controls (unpaired Student’s t-test). (**G**–**L**) 5dpf zebrafish were incubated with hydrolyzed Texas Red (TR, **G**) for 1 hour, gentamicin-conjugated Texas Red (GTTR, **H**) 50 µM for 1 hour, Qx 300 µM for 8 hours +GTTR 50 µM for 1 hour before the end of the experiment (**I**), neomycin-conjugated Texas Red (NeoTR, **J**) 100 µM for 30 min or with Qx 300 µM for 8 hours +NeoTR 100 µM for 30 min before the end of the experiment (**K**). (**L**) The fluorescence intensity incorporated per HC was expressed as a percentage from the corresponding aminoglycoside-Texas Red (AGTR) incubation treatment and represented as mean +/− SEM. Unpaired Student’s t-test. ***p < 0.001. Student’s t-test *versus* corresponding aminoglycoside-only treatment. Scale bar: (**A**–**C**) 6 μm, (**G**–**K**) 5 µm. Data were taken from at least 10 animals and 3 experiments runs. For microphonic potentials 8–9 animals were analyzed per treatment.

There is strong evidence supporting the notion that neomycin, gentamicin, and cisplatin can enter the HCs in a transduction-dependent manner, most likely through the mechanotransduction channels^21,29,35,52,53^. Based on that premise, any agent that blocks the mechanotransduction channels would also block the entrance of ototoxins. We therefore, decided to test whether Qx protects from ototoxic damage by blocking HC mechanotransduction channels. FM1–43 uptake was used as a proxy for mechanotransduction channel activity in zebrafish exposed to E3 media or QX 300 μM for 7 hours. Figure 7A–D shows that rapid dye entry into the HCs was comparable between treatments. Quantification of the fluorescence intensity incorporated by the neuromasts did not show any significant differences (Fig. 7D), indicating that Qx does not block the mechanotransduction channels.

HC mechanotransduction activity was also assessed by microphonic recordings (Fig. 7E,F). For this purpose, 5dpf zebrafish larvae were kept in E3 media alone (control) or Qx 300 μM, and recordings were performed from neuromasts under these conditions. The *pcdh15a* mutant zebrafish line, *orbiter*^54^, was used as a negative control. As shown in Fig. 7E,F, no significant differences were observed in the magnitude of microphonic responses between the controls and Qx-treated animals, suggesting that the HCs remained functional in the presence of Qx. Taken together; these results suggest that Qx does not protect HCs by blocking ototoxin entrance. Instead, it might be acting as a modulator of ototoxins’ harmful effects at the intracellular level.

To test this hypothesis, we conjugated GM and Neo to the dye Texas Red (GTTR and NeoTR, respectively), and analyzed ototoxin incorporation in the presence/absence of Qx 300 µM (Fig. 7G–L). GTTR was incorporated into the HCs independently of Qx. No differences in the fluorescence intensity were observed between GTTR alone or in the presence of Qx (Fig. 7H,I,L). Conversely, in the case of NeoTR, we saw a significant decrease in the fluorescence incorporated per HC when Qx was present (Fig. 7J–L). Since Qx does not block the mechanotransduction channels, this result suggests that in the case of Neo, Qx might be promoting ototoxin extrusion from the HCs.

### Quinoxaline does not interfere with aminoglycoside efficacy

**Figure 8:**
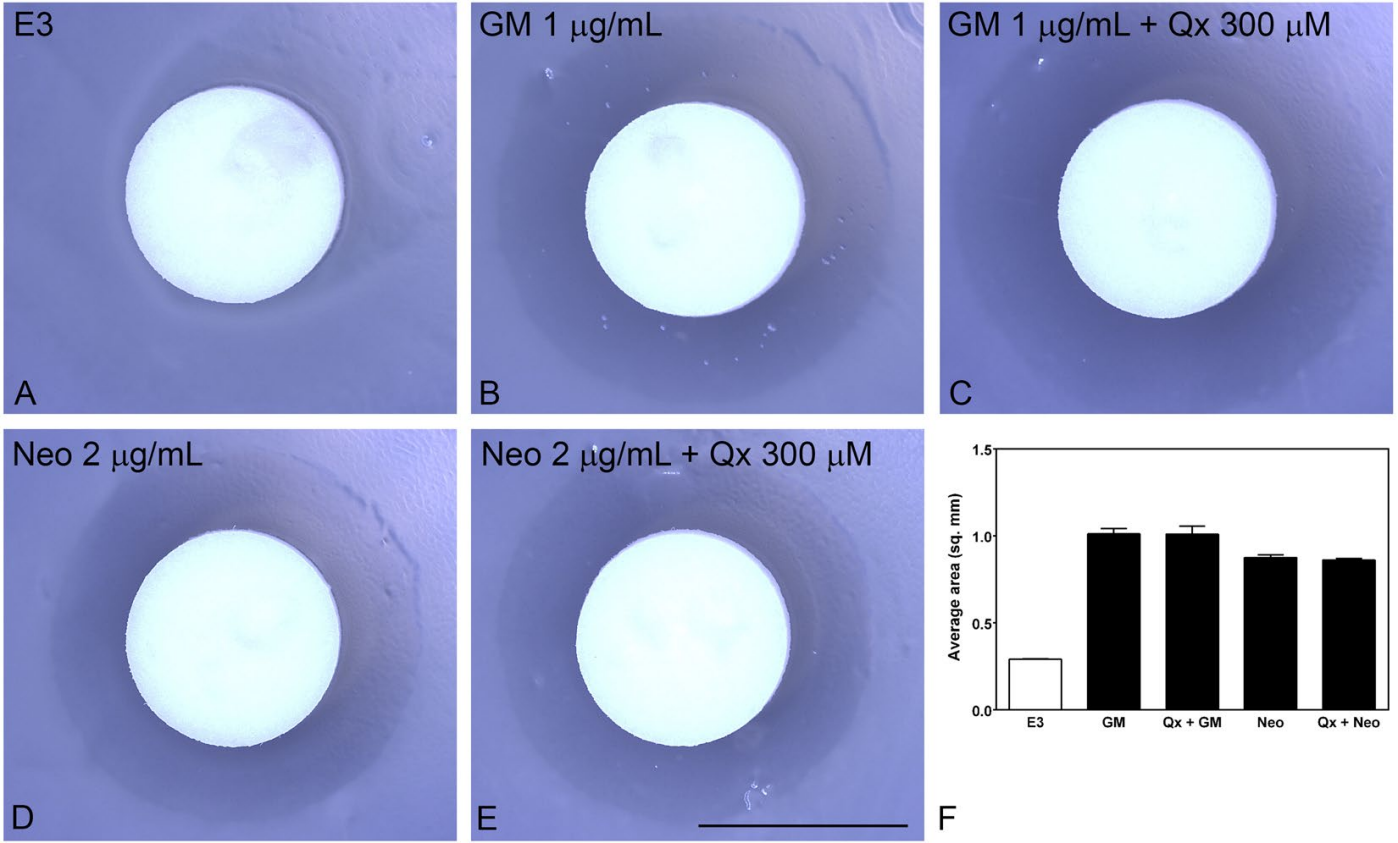
Qx does not affect antibiotic efficacy. (**A**–**F**) No differences were observed in the bacterial-growth inhibitory areas between aminoglycoside-only and aminoglycoside with Qx. Filters were soaked in E3 media (**A**) or in the minimum inhibitory concentration for GM (**B**,**C**) or Neo (**D**,**E**) in the absence (**B**,**D**) or presence (**C**,**E**) of Qx 300 µM. (**F**) Quantification of the inhibitory area expressed as mean +/− SEM. Unpaired Student’s t-test analysis did not show any significant differences between aminoglycoside with or without Qx. Scale bar: 6 mm. Data were taken from 7 replicas and 2 experiments runs.

To determine whether Qx might interfere with aminoglycoside’s ability to inhibit bacterial growth, we performed a diffusion test^22^. *E coli* (strain ATCC25922) were exposed to the minimum inhibitory concentration of each antibiotic (GM 1 µg/mL and Neo 2 µg/mL,^22^) alone or in the presence of 300 µM of Qx and the inhibitory area calculated after overnight incubation (Fig. 8). At the concentration used in our studies, Qx did not affect the ability of aminoglycosides to inhibit bacterial growth (Fig. 8A–E). No significant differences were observed in the presence or absence of Qx (Fig. 8F). Furthermore, no differences were observed in the inhibitory area when Qx was co-incubated with high antibiotic concentrations (data not shown), suggesting a lack of interaction between Qx and gentamicin or neomycin at higher doses.

## Discussion

The results presented here support an otoprotective role for Qx, a quinoline-like ring-containing compound, against the deleterious effects of cisplatin and aminoglycosides. Previous studies suggest that these two types of ototoxins can kill HCs through both distinct and overlapping mechanisms^4,21,23,55^. Mitochondrial dysfunction and HC apoptosis are common processes shared by these ototoxins^4,21,23^. However, aminoglycosides can also induce HC death via caspase-independent apoptosis and necrosis^4^. The activation of Rho GTPases is associated with aminoglycosides’ toxicity while it is known that cisplatin activates the c-jun N-terminal kinase pathway that leads to HC death^4,21,23^. A common feature shared by both, cisplatin and aminoglycosides is the requirement for HC mechanotransduction activity. It has been shown that rapid entrance of these ototoxins requires the direct or indirect involvement of the mechanotransduction machinery^4,20,21,23,29–31,55^. Since multiple pathways can be triggered by these ototoxins, we were not surprised to observe different Qx efficacies in terms of HC protection. On the other hand, given Qx’s broad otoprotective spectrum and the fact that it does not block the mechanotransduction channels, these observations led us to believe that Qx is interfering with a common toxin-induced mechanism^23,25,26,28,32,56,57^. Additionally, Qx might be protecting HCs from dying by increasing the delivery of the ototoxin to lysosomes and/or by increasing its extrusion rate^21,31^. A recent work from Hailey *et al*.^31^ using Texas Red-conjugated aminoglycosides has shown not only that these ototoxins can enter HCs by nonendocytic and endocytic mechanotransduction-dependent pathways, but also that their intracellular sequestration into the lysosomes results in HC protection. Based on this evidence, it is also possible that Qx might be modulating ototoxin intracellular partitioning between the cytosol and the lysosomal compartments and thus, protecting HC from drug-associated toxicity. This can be particularly true for neomycin since we observed a significant reduction in NeoTR when HCs were co-incubated with Qx. Both, cytoplasmic as well as corpuscular NeoTR were reduced in HCs from these animals.

Although it is known that cisplatin kills cancer cells by forming DNA adducts^23,25–27,58^, it is still not completely clear whether a similar process occurs in HCs. What is known is that activation of caspases is an essential event necessary for cisplatin-induced ototoxicity^59–61^ and that its deleterious effect is the result of a cumulative process inside the HCs^23^. Our data are consistent with both, a preventive and therapeutic effect of Qx against cisplatin, since HCs were protected after only two hours of Qx pre-treatment or during Qx co-treatment. The fact that Qx confers otoprotection against CP over a range of concentrations with the concomitant reduction in TUNEL-positive cells, and based on the results obtained with NeoTR, we believe that Qx might be protecting HCs against CP by stimulating its intracellular extrusion.

Several lines of evidence support the existence of distinct aminoglycoside-induced HC death mechanisms^4,15,16,21,62^. For example, neomycin can induce mitochondrial dysfunction via a Bax-dependent process while gentamicin can do the same through a Bax-independent process^4,21,22,55,62^. Moreover, differences in the way each aminoglycoside is accumulated inside the cell have also been observed, making very difficult the identification of a compound that will confer protection against a broad range of aminoglycosides^21,22,31,35^. In our studies, we employed gentamicin and neomycin as examples for acute aminoglycoside ototoxicity. We were not surprised to see that Qx was blocking HC damage with different efficiencies. In the case of neomycin, Qx showed partial protection. Despite the different incubation conditions or different combinations of neomycin and Qx, Qx’s otoprotection against neomycin was always partial. Contrary to neomycin, neuromasts exposed to Qx and gentamicin retained their HCs, giving values similar to control animals. These results reflect the differences observed for aminoglycoside-signaling pathway activation^4,21,22,31,35^ and strongly suggest that Qx is blocking only a sub-group of these pathways. Thus, while for neomycin, Qx might be blocking only a few of several damaging mechanisms, for acute gentamicin exposure, Qx might be inhibiting most of the ototoxin-associated pathways. Furthermore, the pathways that are being affected by Qx may not be shared by both aminoglycosides since the differences observed between GTTR and NeoTR uptakes in the presence of Qx. Additional studies are granted to assess these assumptions.

To test levels of HC death, we performed TUNEL assay for the different treatments. Consistent with Qx’s otoprotective effect, we observed a reduction in the number of TUNEL-positive HCs when animals were co-incubated with the ototoxin and Qx. For cisplatin studies, an improvement in HC number always resulted in a reduction of TUNEL-positive HCs to control levels. However, this was not the case for the aminoglycoside experiments. Although animals exposed to gentamicin and Qx showed an increase in the number of HCs compare with gentamicin alone, TUNEL-positive HCs were still observed. Previous work has shown that gentamicin’s ototoxicity can be divided into at least two phases, a rapid phase occurring over a period of 30 to 90 min and a more slowly phase that continues, even in the absent of gentamicin, for several hours or days^21^. In this light, animals co-treated with gentamicin and Qx followed by 5 hours of recovery did not show any improvement in the number of HCs per neuromast. Moreover, HC number decreased compared to animals co-treated with the two compounds but no recovery. This implies that although gentamicin was removed and neuromasts looked normal, the HCs were still in the process of dying due to the slow gentamicin ototoxic phase. Since we did not observe any TUNEL-positive HC after the 5 hours recovery and because there was still a reduction in the number of HCs, this suggests that gentamicin is promoting HC death by a different process undetectable by TUNEL (i.e., necrosis). Contrary to gentamicin, neomycin ototoxic effect does not go beyond the first 90 min^21^, suggesting that there is no cumulative effect associated with neomycin. In accordance with this, we observed a significant reduction in the number of TUNEL-positive HCs in neomycin-Qx treated animals despite the 40–50% effect in HC protection. These findings not only provide additional evidence regarding the differences between gentamicin’s and neomycin’s mode of action but also suggest that Qx might be only an effective protective compound for acute aminoglycoside-associated ototoxicity.

After damage, zebrafish neuromast HCs can rapidly regenerate and new HCs can easily be confused with protected HCs^36–38^. However, ototoxin-induced HC death occurs within hours while HC proliferation is not evident until two days post-trauma^36,37^. We performed BrdU experiments to confirm the lack of HC proliferation in the presence of Qx with or without ototoxin. As expected HCs were negative for BrdU, however we observed the proliferation of the mantle cells, the HC progenitors^38^. These results agree with previous studies demonstrating supporting cell proliferation over short periods of time after ototoxic damage^22,37^. Similarly, ongoing studies in mice performed by our group showed that Qx has a positive effect in auditory supporting cell proliferation (Patent number: US 9,925,185 B1). This result implicates that Qx is not only protecting HCs against ototoxin but positively influencing proliferation and downstream differentiation of supporting cells into new HCs.

Qx did not interfere with antibiotic activity making it an attractive compound for future therapeutic studies. Moreover, Qx’s lack of protection against GM’s cumulative effect, suggests that it could be more effective against Neo- than GM-mediated ototoxicity, despite the fact that Qx only partially protects HCs from Neo harmful effect. These results have important implications for the treatment and protection of the mammalian sensory hair cells and ultimately for its use as a potential therapeutic drug to prevent and treat hearing loss in humans.

Several studies employing a diverse battery of quinoline-like ring containing compounds have shown that these molecules protect HCs by blocking ototoxin uptake^22,39,63^. The main difference between these compounds and quinoxaline is their size. They are big molecules compared to Qx which explains why they can block ototoxin uptake through the mechanotransduction channels. In the case of Qx, which is a smaller molecule, it can be rapidly incorporated into the HC without affecting the mechanotransduction channel machinery and thus, offering a better alternative to reduce the ototoxic side effects.

In summary, this work describes a novel role for Qx in HC otoprotection against the side effects of cisplatin and aminoglycoside antibiotics. A better understanding of Qx’s mechanism(s) of action will maximize the ability to design more efficient therapeutic drugs that will protect HCs without affecting aminoglycoside or cisplatin efficacy, and thus prevent hearing loss in humans.

## Materials and Methods

### Animals

Zebrafish (*Danio rerio*) experimental larvae of either sex were obtained by pair matings of adult fish maintained at Boys Town National Research Hospital by standard methods approved by the Institutional Animal Care and Use Committee. We used AB wild-type fish, as well as, *Tg(brn3c:GFP)* and *orbiter*^*th263b*^ zebrafish lines^54,64^. The *Tg(brn3c:GFP)* transgenic zebrafish line expresses a membrane-bound GFP in hair cells. *Orbiters* carry a non-sense mutation in the protocadherin-15a gene that results in mechanotransduction impairment. Fish were maintained at 28.5 °C in E3 media (5 mM NaCl, 0.17 mM KCl, 0.33 mM CaCl_2_ and 0.33 MgSO_4_, pH 7.2) **a**t a density of 50 embryos/larvae per 100 mm^2^ Petri. Experimental animals were used at 5dpf and cryoanaesthetized after treatment and before fixation. The following neuromasts were analyzed in our studies: IO4 (infraorbital 4), OP1 (opercular 1), M2 (mandibular 2), O1 (otic 1), O2 and MI2 (middle 2).

Methods were performed in accordance with the guidelines and regulations approved by Boys Town National Research Hospital.

### Drug preparation

Cisplatin (479306), quinoxaline (Q1603), neomycin (N1876) and gentamicin (G1914) were obtained from Sigma. A stock solution of cisplatin 0.5 M was prepared in DMSO and diluted in E3 media to the corresponding working concentrations (50 µM to 800 µM). The 400 µM dose of cisplatin used for most of the experiments was chosen based on previous reports^22,23^. Stock solutions for Qx 1 M, neomycin 100 mM, and gentamicin 50 mg/ml were directly prepared in E3 media. Gentamicin (50–200 μM) and neomycin (50–400 µM) concentration ranges were based on previous reports^21,22,35^. All drugs were dissolved immediately before use.

### Drug treatments

Larvae (15–20 fish) were distributed into transfer baskets and treatments were performed in 24-well plates. Two different approaches were used for cisplatin ototoxicity studies. Protocol 1: to study Qx preventive effect, animals were exposed to Qx (50 μM-300 μM) for 2 hours and then to cisplatin alone for six more hours. Protocol 2: To study Qx therapeutic effect animals were exposed to Qx for 2 hours and then co-treated with Qx and cisplatin for 6 hours. For cisplatin dose-response studies only Protocol 2 was used with cisplatin concentrations ranging from 50µM-800µM.

For the aminoglycosides studies, animals were treated with Qx (50 μM,−300 μM) for 8 hours and the corresponding aminoglycoside added 30 min (for GM) or 1 hour (for Neo) before the end of the experiment. For aminoglycosides dose-response curves, concentrations ranged from 50µM-200µM for GM and 50µM-400µM for Neo. For GM long-term studies, animals were treated as above with 50 µM of GM in the presence/absence of Qx 300 µM and then transferred to fresh E3 media for an additional 5 hours before fixation.

Control animals were exposed to vehicle (DMSO or E3) and included in each experiment. After treatment, animals were transferred to E3 media for 30 min to recover and then fixed with 4% paraformaldehyde (PFA) overnight at 4 °C.

### Hair cell assessment

Immunohistochemistry experiments were performed as described before^65^. Antibodies used in this study: otoferlin (HCS-1, Developmental Studies Hybridoma Bank) and GFP (NB100-1614, Novus Biologicals).

### Neuromast scores

An arbitrary scoring protocol was used to rank the morphology of each neuromast after treatment as follow: 1 (normal rosette-like shape, HCs look normal, hair cell bundles are properly arranged), 2 (normal rosette-like shape, a few HCs appear to be missing, but hair cell bundles are properly arranged), 3 (normal rosette-like morphology but several HCs are gone and hair cell bundles look disturbed), 4 (lthe rosette-like shape is lost in certain areas, few HCs, and few disrupted hair cell bundles), 5 (No rosette-like shape, one or two disperse hair cells and no hair cell bundles). At least 25 animals per treatment were assessed. The assessment was performed double-blind and unbiased. Scores were expressed as percentages of the total of neuromasts analyzed per treatment.

### Terminal deoxynucleotidyl transferase dUTP nick end labeling assay

The TUNEL assay was performed employing the *in situ* cell death detection kit, TMR red (12156792910, Roche) and according to the manufacturer’s instructions^65^.

In the case of cisplatin TUNEL studies, animals were pre-incubated with Qx 300 μM for 2 hours and then co-incubated with Qx and cisplatin 400 μM for two additional hours. This incubation approach was chosen because we noticed that when assessing TUNEL after 4–6 hours with cisplatin, the HCs were far beyond the DNA fragmentation step, resulting in TUNEL-negative HCs (the few HCs left after ototoxin treatment). Shortening the exposure time improved the TUNEL results and reduced variability.

### Cell proliferation assay

Cell proliferation experiments were conducted using bromodeoxyuridine (BrdU) according to Kruger *et al*.,^22^. Immunohistochemistry for BrdU was performed according to He *et al*.^66^.

### Aminoglycoside-Texas Red conjugation and uptake experiments

GM and Neo were conjugated to Texas Red as previously described^53,55^. Briefly, GM or Neo and Texas Red succinimidyl ester (Thermo Fisher) were incubated overnight at a molar ratio 3:1 to produce the corresponding conjugate solution. The labeled aminoglycosides were diluted in E3 media to the final working concentrations (50 µM for GM and 100 µM for Neo), and incubations were performed in the presence/absence of Qx 300 µM as described above. As control animals were incubated in E3 media containing hydrolyzed Texas-Red for 1 hour.

For FM1-43FX (Thermo Fisher) uptake, experiments were performed as previously described^67^. Briefly, animals were incubated with E3 alone or in the presence of Qx 300 μM for 7 hours and then immediately exposed to 3 μM of FM1-43FX for 40 sec or animals were transferred to a fresh E3 solution for 1 hour and then exposed to the dye.

In all the experiments, the fluorescence incorporated was quantified according to Ogun & Zallocchi^67^ using ImageJ.

### Microphonic potentials

Mechanotransduction was assayed by performing extracellular recordings of microphonic responses from neuromasts. Details for recording microphonic potentials have been provided before^68,69^. In brief, zebrafish were embedded in agarose and mounted in the experimental chamber. A stimulus microprobe with a tip diameter of 3 μm was positioned 5 µm near a neuromast. The displacement of the microprobe, driven by a piezoelectric actuator (Burleigh Driver/Amplifier, PZ-150M), was calibrated by a photodiode-based system^70^. The displacement and orientation of the probe after the probe was positioned were kept constant for all recordings. Sinusoidal bursts with frequencies of 100 or 200 Hz were used. The displacement of the probe was set at approximately 3 μm, sufficient to generate maximum (saturated) microphonic potentials.

Patch electrodes were used to record microphonic potentials. The recording electrode had open tip resistances of approximately 5–7 MΩ when filled with standard fish saline solution (29.6 mM NaCl, 2.7 mM KCl, and 1.8 mM CaCl_2_ with pH of 7.2). The microphonic responses (filtered at 1 kHz) were amplified using an Axopatch 200B amplifier and acquired using pClamp 9.1 (Axon Instruments) running on an IBM-compatible computer with a 16-bit A/D converter (Digidata 1322B). Twenty averages were preset for each recording. Data were analyzed using Clampfit in the pClamp software package and Igor Pro (WaveMetrics, Inc).

### Aminoglycoside efficacy studies

Aminoglycoside efficacy tests^22^ were used to address whether Qx otoprotection affects the ability of GM or Neo to inhibit bacterial growth. Briefly, filter discs were soaked in E3, aminoglycosides or aminoglycosides and Qx 300 µM. The lowest effective aminoglycoside concentration was used (1 µg/mL for GM and 2 µg/mL for Neo) to test if Qx interferes with the antibiotic efficacy^22^. To rule out possible Qx/aminoglycoside interactions at higher aminoglycoside dilutions, 10,000 × aminoglycoside concentrations were also tested. *E. coli* (strain ATCC25922) was plated in agar plates and the filter discs lean on top of them. Agar plates were incubated overnight and imaged using a Leica MZ10 microscope. The inhibitory area was calculated for each condition and expressed as square mm.

### Microscopy

Zebrafish confocal images were captured at RT using a Zeiss LSM 800 confocal microscope. Z-stack images were acquired using the 63×, NA 1.4 oil objective at a 2× zoom, and with sectioning set automatically to optimal. Images were acquired and processed with ZEN 2 black edition software (Carl Zeiss). Z-stack images are presented as flat Z-projections. Only linear adjustments were made to brightness and contrast, and the final figures were assembled using Photoshop and Illustrator software (Adobe).

### Data analysis and statistics

For each treatment, 10–25 animals were used, and the experiments were repeated at least three times. At least three neuromasts per fish were assessed. Results were presented as mean ± SEM. Microphonic potentials were performed in 8–9 animals and presented as mean ± SD. For antibiotic efficacy tests seven inhibitory areas were measured for each treatment, averaged and statistically analyzed.

Statistical analysis was performed using Prism 5 (version 6.07) Comparisons were made by one-way ANOVA followed by Dunnett’s multiple comparisons test or by two-tailed Student’s t-test. Ototoxin alone and ototoxin + Qx treatments were compared to control (statistics in black). Ototoxin + Qx treatments were compared to the corresponding ototoxin alone (statistics in red).

Animals treated with vehicle alone or with the different Qx concentrations did not show any differences in the number of HCs, neuromast scores or TUNEL and were averaged together and considered as controls.
