# Author Correction: Quinoxaline protects zebrafish lateral line hair cells from cisplatin and aminoglycosides damage

**DOI:** 10.1038/s41598-019-44366-1

**Published:** 2019-06-14

**Authors:** Sonia M. Rocha-Sanchez, Olivia Fuson, Shikha Tarang, Linda Goodman, Umesh Pyakurel, Huizhan Liu, David Z. He, Marisa Zallocchi

**Affiliations:** 1 School of Dentistry, Creighton University, Omaha, NE 68178 USA; 2 Boys Town National Research Hospital, Omaha, NE 68131 USA; 3 Department of Biomedical Sciences, Creighton University School of Medicine, Omaha, NE 68178 USA

Correction to: *Scientific Reports* 10.1038/s41598-018-33520-w, published online 11 October 2018

The original version of this Article contained a typographical error in the spelling of the author Sonia M. Rocha-Sanchez, which was incorrectly given as Sonia M. Rocha Sanchez. This has now been corrected in the PDF and HTML versions of the Article.

